# New Phase of Growth for Xenogeneic-Based Bioartificial Organs

**DOI:** 10.3390/ijms17091593

**Published:** 2016-09-21

**Authors:** Zorina Pitkin

**Affiliations:** Organogenesis Inc., 150 Dan Road, Canton, MA 02021, USA; zpitkin@organo.com; Tel.: +1-781-401-1009

**Keywords:** bioartificial organs, xenotransplantation, gene editing, clinical trials, risk assessment

## Abstract

In this article, we examine the advanced clinical development of bioartificial organs and describe the challenges to implementing such systems into patient care. The case for bioartificial organs is evident: they are meant to reduce patient morbidity and mortality caused by the persistent shortage of organs available for allotransplantation. The widespread introduction and adoption of bioengineered organs, incorporating cells and tissues derived from either human or animal sources, would help address this shortage. Despite the decades of development, the variety of organs studied and bioengineered, and continuous progress in the field, only two bioengineered systems are currently commercially available: Apligraf^®^ and Dermagraft^®^ are both approved by the FDA to treat diabetic foot ulcers, and Apligraf^®^ is approved to treat venous leg ulcers. Currently, no products based on xenotransplantation have been approved by the FDA. Risk factors include immunological barriers and the potential infectivity of porcine endogenous retrovirus (PERV), which is unique to xenotransplantation. Recent breakthroughs in gene editing may, however, mitigate risks related to PERV. Because of its primary role in interrupting progress in xenotransplantation, we present a risk assessment for PERV infection, and conclude that the formerly high risk has been reduced to a moderate level. Advances in gene editing, and more broadly in the field, may make it more likely than ever before that bioartificial organs will alleviate the suffering of patients with organ failure.

## 1. Introduction

Since the 1960s, researchers have been developing bioartificial alternatives to allotransplantation. The case for their clinical development is clear: allotransplantation is the only remedial solution to organ failure, there is an acute shortage of viable organ transplants, and broad adoption of bioartificial/bioengineered organs would help alleviate it. Bioartificial organs can serve either as a bridge to allotransplantation or organ regeneration, or as independent, implantable units.

Demand for allotransplantation, very successful since the early 1980s, has far outpaced supply [1] and Orlando et al. estimate that they will probably never be brought into balance [2].

**Figure 1:**
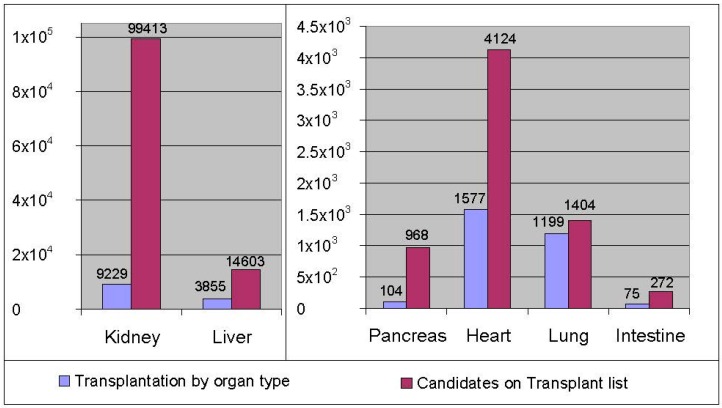
Transplantation procedures for various organ types performed in January–June 2016 in comparison to the number of candidates on the transplantation list as of 29 July 2016. The disparity between organ supply and demand is striking, particularly for kidney, liver, and pancreas. Data adopted from UNOS [1].

Figure 1 illustrates the disparity between the number of patients waiting for transplantation, and the number of transplantations actually performed in the first six months of 2016. The current number of unserved kidney candidates is about 100,000, followed by about 15,000 patients waiting for a liver transplant. Between January and June 2016, 9229 kidney transplants and 3855 liver transplants were performed [1].

Dialysis, pioneered by Koff in the mid-20th century, inaugurated the development of artificial organs [3,4]. Subsequent to the artificial kidney, other artificial organs like the artificial liver with the use of plasma exchange and albumin dialysis have been introduced in the market to address the donor organ problem [5,6]. These systems only provide temporary mechanical support and rely on the processes of adsorption and filtration without replicating the biological functions of the failing organ. Bioartificial organs, in contrast, closely mimic human organs and replace the metabolic functions of the failing organ. This is achieved by integrating a biological component (human or non-human cells and tissues) with synthetic or natural platforms.

These synthetic and natural platforms exist along a broad spectrum of technologies encompassed by the field of regenerative medicine. They are discussed further in subsequent sections. The spectrum includes cellular therapies utilizing allogeneic, autologous or xenogeneic cells; technologies reliant on cells that are integrated into extracorporeal or implantable devices; and autologous cells seeded onto decellularized scaffolds of human, synthetic or animal origin.

For reasons we will review, most bioengineered tissues still remain far from commercialization. It is very difficult to demonstrate safety and efficacy and successfully pass regulatory scrutiny for most bioartificial organs under development, and it is uncertain when these systems will become commercially available [2]. Currently, there are only two active clinical studies involving bioartificial organs [7].

Several comprehensive reviews have been published on bioartificial organs currently in development. They span systems utilizing autologous cells [2,8,9], bioartificial systems at the stage of pre-clinical investigations [10,11], and clinical case reports. This paper will summarize them briefly, and focus on bioartificial organs that have either been approved for clinical investigation or have already been tested in clinical trials. The types of systems reviewed will include those that combine living cells engineered into scaffolds and membrane-based devices. We will focus on systems that utilize xenogeneic cells because of their promise, as they have attained an advanced stage of clinical development, and because of practically unlimited supply of source cells/tissues. As an illustrative case study, we will offer a risk assessment of potential infectivity of porcine endogenous retrovirus (PERV).

## 2. Bioartificial Organs on the Market

Only two bioengineered products, both developed to address injuries to the skin, have had commercial success. Several hundred thousand patients have been treated with the living skin substitutes called Apligraf^®^ and Dermagraft^®^, both manufactured by Organogenesis Inc. (Canton, MA, USA). Apligraf is a living three-dimensional bi-layered tissue derived from neonatal foreskin allogeneic cells. It consists of an epidermal layer formed by human keratinocytes and a dermal layer composed of human fibroblasts incorporated into a bovine collagen matrix. Dermagraft is a living skin substitute derived from neonatal foreskin allogeneic cells, where human fibroblasts are grown on a bio-absorbable mesh scaffold. Both Apligraf and Dermagraft have been extensively tested in controlled randomized clinical trials to demonstrate safety and efficacy and were approved by the Food and Drug Administration (FDA) as class III medical devices through the pre-market approval (PMA) process. Apligraf and Dermagraft are approved for treatment of non-healing wounds in diabetic foot ulcers (DFU) [12,13] and Apligraf is approved for the treatment of venous leg ulcers (VLU) [14]. Since the FDA’s approval of Apligraf for VLU in 1998 and subsequent approval of Apligraf and Dermagraft for DFU, over a million units of Apligraf and Dermagraft have been distributed. A recent scientific report from the University of Miami on the randomized clinical trial shed light on how Apligraf affects patient’s genomic profile when applied to a chronic (non-healing) VLU. The analysis showed that Apligraf altered specific molecular and cellular responses in the wound environment, reversing the chronic wound profile to resemble more of an acute profile. This study provided valuable insights on Apligraf’s mechanism of action [15].

No other bioartificial organs currently in development have received regulatory approval for commercial distribution. Clearly, there remain barriers to implementing bioartificial organs into patient care that first surfaced 20–30 years ago.

## 3. Representative Examples of Bioartificial Organs

A proposal was recently published with a four-pronged strategy to address organ shortage [16]. The strategy calls for: (1) organ bio-printing; (2) scaffold re-cellularization; (3) optimization of cellular repair and regeneration; and (4) xenotransplantation.

Bioartificial organs continue to be the technology of choice to bring organ supply in balance with demand. A few examples of bioartificial organs follow, categorized as in [16]. Some of these systems have been tested clinically, and others are in the earlier stages of research and development. Unfortunately, successes in initial clinical studies are not necessarily predictive of results of controlled clinical trials. Moreover, outcomes of clinical investigations of the bioartificial organs described in this paper are quite pessimistic—none of them have reached commercialization. The outcomes must still be discussed, as both positive and negative results of clinical research will provide a foundation for improved systems that will one day be translated into controlled clinical trials. Systems that have advanced furthest in clinical development are described in more detail.

### 3.1. Organ Bio-Printing

Organ bio-printing is a technology that has been in development over the past two decades. It involves a layer-by-layer computer-aided bio-fabrication of functional 3D organ constructs using self-assembling tissue spheroids [17]. Once created, this living construct would then be implanted into the patient to replace functions of the failing organ. Application of 3D bio-printing has been reported by Atala and his team at the Wake Forest Institute for Regenerative Medicine for the generation and transplantation of heart tissue [18]. It has also been used for multilayered skin, bone, vascular grafts, tracheal splints, and cartilaginous structures. Companies like Organovo, BioBots and others attempt to print 3D living tissue for potential organ replacement. Early research in this field has yet to be translated into the clinical setting.

### 3.2. Scaffold Re-Cellularization

In scaffold re-cellularization, three-dimensional scaffolds are used to create an extracellular matrix (ECM) that mimics the mechanical and geometrical properties of the failing organ. These decellularized matrices, which preserve the architecture of the organ, are then re-cellularized by being seeded with autologous cells to create an autologous organ. Extensive research is ongoing to create complex parenchymal bioartificial organs, including the kidney, liver, pancreas, lung and heart, by creating re-cellularized scaffolds as a platform for seeding with different types of cells [9].

Orlando et al. reviewed the progress in preclinical research in cell-based technologies integrated with decellularized scaffolds as applied to solid organ transplantation [8]. Clinical case studies were reported in 160 patients, 55 of whom received bioartificial organs made from autologous cells that were seeded on scaffolds of various origins [2,8]. These studies were mainly related to bioengineering of urogenital tissues, upper airways, and blood vessels. Decellularization/recellularization of complex organs like the kidney or liver presents a greater challenge that must be resolved. A new approach, termed semi-xenotransplantation, has been recently introduced by Salvatori et al. [19,20]. The authors suggested that porcine ECM scaffolds can be recellularized with patient-derived cells, thus ruling out the need for immunosuppression while removing all antigenic cellular components from the porcine ECM. While human clinical trials are still far off, the development of these bioartificial organs holds much promise.

#### 3.2.1. Bioartificial Bladder

Atala and his team bioengineered bladders using scaffold-seeding technology [21,22]. Bladders were grown on biodegradable scaffolds, seeded with autologous cells and transplanted in seven patients with end-stage bladder disease requiring cytoplasty. Vexingly, clinical studies with the bioartificial bladder did not demonstrate safety and efficacy; still they provided knowledge that will help in the future development of bioartificial organs.

#### 3.2.2. Bioartificial Trachea

Airway bioengineering was applied to several patients across the US, Europe and Russia, who received either a donated or a synthetic trachea seeded with the autologous stem cells [23,24,25]. The results of these clinical case studies, albeit surrounded by controversy [26], may provide important input into future clinical development under controlled clinical trial requirements for bioartificial airways.

### 3.3. Optimization of Cellular Repair/Regeneration

The aim of organ support therapies is to either prevent organ failure or to allow time for the regeneration of native organ function. The aim of the development of the two bioartificial organs that are described next—the bioartificial kidney and bioartificial liver—is to address the largest need in organ transplantation (see Figure 1), where the kidney and the liver are in highest demand. The goal of the bioartificial kidney is to restore native kidney function and, ultimately, to improve survival. The aim of the bioartificial liver is to bridge patients with liver failure to liver transplantation or to allow time for the recovery of native liver function and thus avoid liver transplantation. While some of the bioartificial kidney and liver systems have already been evaluated in controlled, randomized, multi-center clinical trials, none of them have been approved by the FDA for clinical use. Conclusive clinical trials are required to establish safety and efficacy of these much needed bioartificial systems.

#### 3.3.1. Bioartificial Kidney

**Figure 2:**
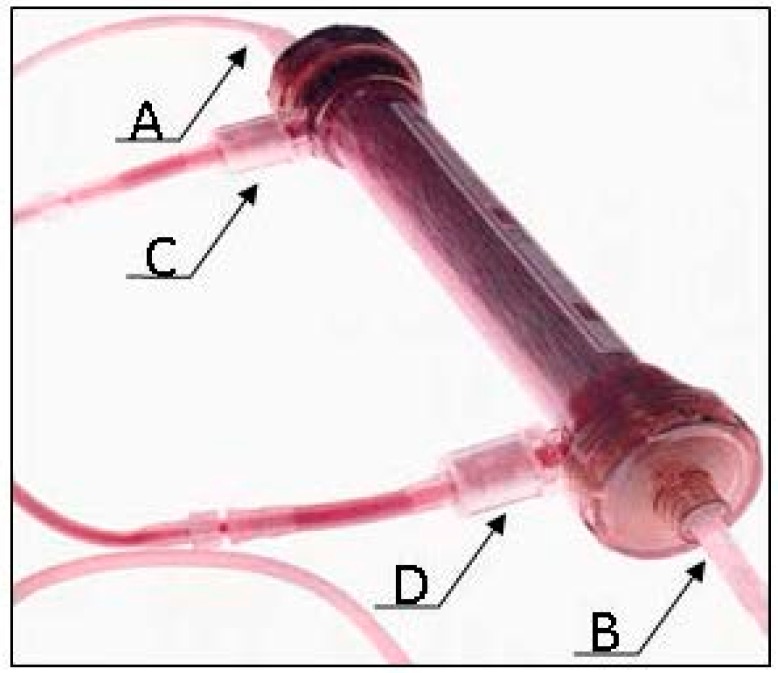
Renal Assist Device (RAD), containing human renal tubule cells (RTC) is part of the two circuit system: a standard hemofilter and a bioreactor (RAD). The ultrafiltrate produced by the hemofilter enters the RAD lumen (A) upon which the RTC have been grown, and then discarded (B); The blood from the hemofilter enters the extracapillary space of the hollow fiber cartridge (C); in the RAD, the blood is separated from the RTC by the semipermeable hollow fiber membrane and returned to the patient (D).

The FDA has allowed only one bioartificial kidney to be evaluated in human clinical trials. The renal assist device (RAD), developed by Humes [27,28], RenaMed Biologics, Inc. (Lincoln, RI, USA), was an extracorporeal treatment system utilizing a standard hemofiltration cartridge containing approximately 10^9^ renal tubule cells (RTC) grown along the inner surface of the fibers. The RAD was seeded with the RTC derived from human kidneys not suitable for transplantation, mainly due to anatomical defects, and cells were expanded in a culture medium [11,29]. The hollow fibers provide support for the cellular system, allow for the transport of essential cell products and nutrients, and prevent the cells from entering the circulatory system. The RAD Circuit consisted of two perfusion loops. The first one was the Continuous Veno-Venous Hemofiltration (CVVH) loop, which is a conventional CVVH system. The second was the RAD loop, which contained the RAD cartridge (Figure 2). During the RAD treatment, blood from the patient was perfused through a conventional hemofilter, which separates the blood into an ultrafiltrate component and a blood cellular concentrate. A portion of the ultrafiltrate component entered the RAD loop and was perfused through the lumen of the RAD cartridge. Within the RAD cartridge, the ultrafiltrate came into direct contact with the RTC attached to the lumenal wall of the hollow fibers. The blood cellular concentrate circulating around the outside of the hollow fibers was separated from the RTC by a semipermeable hollow fiber membrane, through which only small molecular weight molecules contained in the ultrafiltrate or synthesized by the renal tubule cells can pass. Upon exiting the RAD cartridge, the RAD-treated blood cellular concentrate was recombined with the blood cellular concentrate in the CVVH loop and was then returned to the patient.

The RAD was initially evaluated in a 10-patient Phase I/II study in patients with acute renal failure (ARF) due to acute tubular necrosis (ATN) [30]. Based on the results of this clinical study, a Phase II, multicenter, randomized, controlled, open-label trial involving 58 patients with ARF was conducted. Forty patients received CVVH and RAD, and 18 received CVVH alone. The trial demonstrated a statistically significant advantage of RAD with respect to survival as compared to the CVVH group [31]. Unfortunately, these results were not reproduced in the follow-up Phase IIb study.

Other technologies, like an implantable RAD based on microelectromechanical systems, and the transplantable bioengineered kidney based on biological templates seeded with cell lines [20] hold much promise. At present, while they are in the research and development phase, they are far-off from bringing a clinically available bioartificial kidney to market.

Human clinical trials for the bioartificial kidney have not been initiated or re-initiated. Even in the best case scenario, it will take several years to address technical issues related to cell source and cell viability/functionality, resolve safety issues and manufacturing challenges, meet regulatory expectations, conduct well-designed clinical trials, and secure continuous funding to make these bioengineered systems the standard of care for patients requiring kidney transplantation.

#### 3.3.2. Bioartificial Liver

Nyberg [32] summarized over 30 different cell-based liver support devices that have been reported since 1987. More than 14 systems have been evaluated in clinical trials for their capacity to provide liver functions [6,10,33]. Although primary human hepatocytes would seem to be the cells of choice in a bioartificial liver, the availability of these cells is limited. As an alternative, immortalized cell lines of the C3A human hepatoblastoma line have been used in a bioartificial liver device. Primary porcine hepatocytes, which are readily available, have been used in all bioartificial liver systems, with the exception of the Extracorporeal Liver Assist Device (ELAD), which employs the C3A cell line. Despite much progress in understanding the mechanism of action of such systems, none of them have even been reviewed for marketing approval by the FDA. Before progress toward commercialization can be made, multiple variables have to be addressed: the optimization of clinical trial design, the maintenance of cell viability, resolution of regulatory issues, risk mitigation related to xenozoonosis, and outstanding technological challenges.

ELAD, Vital Therapies, Inc. (San Diego, CA, USA) is an extracorporeal system that utilizes C3A cells [34]. The cells are placed in the extracapillary space of a modified dialysis cartridge. Safety mechanisms are in place to prevent tumor cells from entering the patient’s blood stream [35]. The system has been evaluated in several clinical studies in patients diagnosed with Acute Liver Failure (ALF) [36] and in a Phase III clinical trial in subjects with Alcohol-Induced Liver Decompensation (AILD). Studies in both indications failed to achieve their primary and secondary endpoints. Another Phase III clinical trial on Acute Alcoholic Hepatitis (AAH) is planned based on a post-hoc analysis of the AILD trial. Many years in development, the ELAD system remains in the clinical development stage, still far from entering the marketplace.

**Figure 3:**
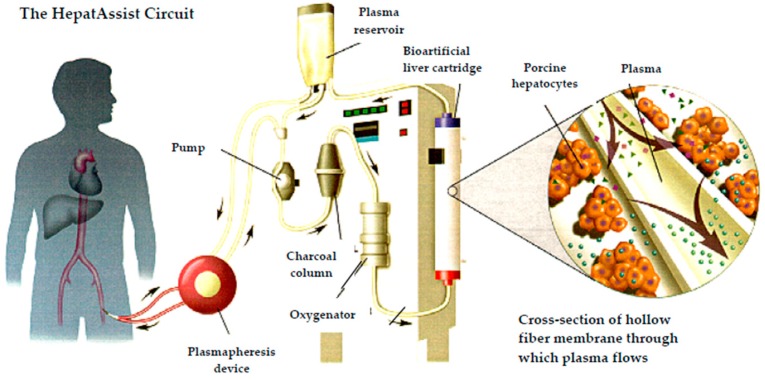
The hepatAssist liver support system.

HepatAssist™, developed by Demetriou et al. [37], Circe Biomedical (Lexington, MA, USA) was the first bioartificial liver assist device tested on a large clinical scale in a Phase II/III clinical trial. The device was comprised of porcine hepatocytes cryopreserved until the cells are thawed and placed in an extracorporeal system in the extracapillary space of a hollow fiber membrane. There was no direct contact between the patient’s plasma and porcine cells during the therapy (Figure 3) [37]. The cells were derived from pigs housed in a specific pathogen-free herd under strict controls and in accordance with regulatory requirements and Circe’s quality program. Cryopreservation allowed for complete microbiological assessment including testing for adventitious agents, and for confirmation of cell viability and relevant functionality prior to clinical application [38]. The hepatocytes in the device performed many of the metabolic functions of a healthy liver. The membrane had been optimized to maximize transmission of key proteins and to minimize transmission of adventitious agents. The system was first investigated in a Phase I study [39], which yielded encouraging results on the potential efficacy of the system. It was further evaluated in the first prospective, randomized, controlled Phase II/III multicenter trial conducted in 19 centers across the US and Europe and which included 171 patients with ALF and primary non-function following liver transplantation. Of these, 86 patients were enrolled in the treatment group with HepatAssist system. This was the largest study in the field of liver support. The system demonstrated safety and improved survival in a subgroup of patients with fulminant/sub-fulminant hepatic failure [40]. To address potential infectivity of PERV, its infectivity was assessed in 103 patients treated with the HepatAssist system in two multicenter clinical trials demonstrating no evidence of PERV transmission [41,42]. As the significant survival benefit was identified only in a post hoc subgroup analysis, the HepatAssist device was not approved by the FDA. Further clinical trials required by FDA to proceed to marketing application could not be conducted due to the financial collapse of the system’s developer.

A hybrid liver support system employing porcine hepatocytes with extracorporeal plasma separation and bioreactor perfusion in patients with ALF was developed by Gerlach [43]. Four separate capillary membrane systems, each forming independent compartments, are woven in order to create a three dimensional network. The bioreactors contained primary hepatocytes obtained from specific pathogen-free pigs. The bioreactor was integrated into a modular extracorporeal liver support (MELS) system, combining biologic liver support with artificial detoxification technology. Development of the MELS system included an eight-patient clinical study [44]. No PERVs were detected in patients treated with the system [44,45]. Despite initial encouraging results, the system never progressed to a prospective, controlled, randomized, clinical trial required for regulatory approval; consequently, the system never reached the marketplace.

A new bioartificial liver assist system called the Spheroid Reservoir Bioartificial Liver (SRBAL) has been developed by Nyberg at Mayo Clinic [46] as a long-term treatment option to liver transplantation. The SRBAL utilizes primary porcine hepatocytes within three-dimensional spheroid aggregates formed by cell-to cell adhesion mediated by surface molecules. The spheroid structure is thought to protect hepatocytes from apoptosis. The SRBAL is incorporated into an extracorporeal circuit. The system was recently evaluated in a prospective randomized controlled translational study in an ALF animal model. One of the important findings of the study was that the SRBAL maintains functionality of primary pig hepatocytes in the range of normal liver physiology. Further clinical studies that are planned to evaluate the SRBAL system will be the first re-initiation of clinical development of extracorporeal membrane-based bioartificial livers with pig cells after a long period of stasis in this field (see discussion on xenotransplantation that follows).

Lessons learned from using these extracorporeal liver assist systems intended to treat liver failure of various etiologies may guide future research and development of a bioartificial liver that can finally reach patients as an approved therapy.

## 4. Bioartificial Organs and Xenotransplantation

Xenotransplantation is defined by FDA as “any procedure that involves the transplantation, implantation, or infusion into a human recipient of either (a) live cells, tissues, or organs from a nonhuman animal source; or (b) human body fluids, cells, tissues or organs that have had ex vivo contact with live nonhuman animal cells, tissues or organs” [47]. Its modern history dates back to the beginning of the 20th century [48]. Due to the fast rejection of the grafts and limited knowledge of immunology, early attempts at xenotransplantation were unsuccessful. With advances in immunology in the second half of the 20th century, interest in xenotransplantation was rekindled and experiments were restarted in a limited clinical setting [49]. Xenotransplantation from non-human primates, once the dominant source, was no longer the focus of research, mainly due to ethical and microbiological issues.

Among discordant species that can potentially be used for xenotransplantation, pigs offer a plentiful source of tissues and organs. In addition, pig organs are comparable in size and are physiologically similar to human organs. Unlike the use of non-human primates, the use of pig species for organ transplantation raises little or no ethical concerns because of their acceptability as a food source and well-established pig husbandry practices.

Based on the FDA’s definition, we refer to all bioartificial organs that employ pig cells or tissues as “xeno BAO”. Important knowledge has been gained through clinical development of xeno BAO. Examples include the HepatAssist liver support system [40], MELS [44], encapsulated pig islet cells [50], and others. Xeno BAO systems have been plagued neither by adverse immunological reactions nor by PERV infection. Methodologically, clinical trial experience has helped optimize study design, and also to improve delivery of bioartificial organs to distant clinical sites.

### 4.1. Risks in Xenotransplantation

The potential benefit of xenotransplantation has to be evaluated against risks associated with the procedure. Since pigs are considered the species of choice in xenotransplantation, we will briefly review the risks associated with the use of bioartificial organs sourced from pig organs/tissues. The two risk criteria we will use are immunological barriers and xenozoonosis. Xenozoonosis encompasses microbiological risks including the risk of cross-species transmission of PERV.

#### 4.1.1. Immunological Barriers

Acute rejection is observed both in allogeneic and xenogeneic-based therapies. One of the main barriers to successful xenotransplantation is hyperacute rejection (HAR) of the graft, which is characterized as a loss of function of the transplanted organ and is specific to xenotransplantation [51]. Current approaches to avoid such rejections intend to reduce or eliminate immunological risks [52].

Over the past two decades, genetically engineered pigs have been introduced to circumvent HAR. These include pigs with human complement-regulatory protein expression and pigs in which the gene for α1,3-galactosyltransferase has been knocked out (GTKO pigs) to alleviate the humoral immunological barriers [53]. Recent studies have demonstrated the feasibility of multiplex genetic engineering [54,55]. The latest innovations include the use of clustered regularly interspaced short palindromic repeat/CRISPR-associated protein 9 (CRISPR/Cas9) in production of GTKO pigs [56]. Efficiency in the pig genome editing has recently been achieved by Tector’s team [56].

The advantages offered by genetic engineering to obviate the human immune response may ultimately address the organ shortage [57]. This technology is expected to move xenotransplantation forward more rapidly [54,58]. Cooper et al. commented that “it is likely, therefore, that more rapid progress will be achieved in the genetic manipulation of pigs for the specific purposes of xenotransplantation” [59]. Genome editing by CRISPR/Cas9 in combination with induced pluripotent stem cells (iPSC), may enable the creation of human organs from genetically-modified chimeric pigs [60].

Research advancements in the development of transgenic pigs will potentially eliminate challenges and concerns related to overcoming the immunological barriers [61]. However, despite notable achievements made in the past two decades in generating transgenic pigs [62], the risk of xenotransplantation in the medical practice remains a hope to be realized in the future.

In addition to risk mitigation through genetic engineering of pigs for organ transplantation, the use of extracorporeal permselective membrane-based xeno BAO and encapsulated pig cells significantly reduce the risk of immunological reaction and tissue/cell rejection.

#### 4.1.2. Risk of Xenozoonosis

There is a risk of transmission of both known and unknown infectious agents from pig to human. Risk mitigation techniques include using robust characterization of pigs from specific pathogen-free herds, employing strict controls of pig husbandry, screening for infectious agents, control of animal feed, and maintaining necessary containment, as promulgated by FDA requirements [47]. Among viral pathogens that have been identified as the most concerning for xenotransplantation, due to their potential to be zoonotic, are PERV, porcine cytomegalovirus (PCMV), porcine lymphotropic herpes virus (PHLV), Hepatitis E (HEV), and porcine circovirus (PCV). State-of-the-art detection methods for viral pathogens allow for a careful screening for adventitious agents, for surveillance of the herd [63], and for quality control of the cells or tissues derived from the pig [38].

Concerns have been raised that the use of pig organs, tissues and cells may facilitate the cross species transfer of infectious agents such as PERV. In the late 1990s, clinical development of xenotransplantation was hindered by the discovery of PERV capable of infecting a human cell line in vitro [64]. This finding raised considerable safety concerns about the clinical use of tissues and cells sourced from pigs. PERVs raised a new kind of regulatory, scientific and public fear because they are integrated in the genome of all pigs and are able to infect human cells under laboratory conditions. As a result, clinical research slowed down, research funding dried up, and the prospect of xenotransplantation becoming a reality diminished. The risk of the unknown dominated the field, especially the risk to a broader human population. The focus of xenotransplantation research turned to strategies to mitigate the microbiological risks of xenotransplantation [47,65]. In particular, research focused on addressing PERV infectivity issues to human recipients [66].

The World Health Organization (WHO) and the FDA have held a number of public hearings and expert consultations on the subject of regulatory requirements for xenotransplantation and, in particular, regarding PERV infectivity to the individual patient and to public at large [47,67,68]. The International Xenotransplantation Association has recently published a consensus statement on conditions for undertaking clinical trials regarding potential transmission of all porcine microorganisms [69], which followed a previously issued consensus focused on strategies to prevent transmission of PERV. Based on the analysis, it was proposed that monitoring of patients for certain clinically relevant microorganisms would not be required so long as these microorganisms are included in pig screening programs.

There has never been a documented case of a PERV infection in any human recipient of porcine-derived organs, tissues or xeno BAO. However, risk mitigation of PERV transmission is necessary to address regulatory concerns, as the FDA is still concerned about the possibility of disease caused by PERVs [70].

#### 4.1.3. PERV Infection: Risk Assessment

As PERV has been one of the principle barriers to the acceptance of xenogeneic-based bioartificial organs, we present a risk assessment of PERV infection and the acceptability of risk relative to patient safety. Our approach includes the measurement of: (a) severity of the event; (b) detectability; and (c) likelihood of event occurrence in relationship to patient safety. The complex issues facing xenotransplantation and bioartificial organs related to PERV transmission can be analyzed from this relatively straightforward perspective.

The risk identification process begins with the analysis of recognized risks that have been observed previously as well as an assessment of unrecognized or unforeseen risks. By means of applying a qualitative scale for risk assessment described in ICH Q9 “Risk Assessment” [71], the risks of PERV transmission in xenotransplantation can be compared based on: (1) data available by the time of discovery of PERV cross-species infection (circa 1997); and (2) currently available PERV infectivity data.

A risk level is assigned based on Severity, Detectability and Likelihood of occurrence. Severity is defined as a measurement of the possible consequences of a hazard; detectability is the probability that the effect of the failure will be detected; and likelihood is defined as the frequency with which the failure occurs.

##### Severity

The potential consequences of PERV transmission to the individual patient and public at large were carefully reviewed by FDA, WHO, and experts around the world following the discovery of PERV transmission to the human cell line in vitro. The severity of PERV infection was characterized as “high”, especially because this type C virus is found in the genome of all pigs. In 1997, all clinical trials involving xenotransplantation in the US were placed on clinical hold based on the well-founded fear of PERVs’ inducing severe immunosuppression. Sponsors responded actively to the potentially emerging issue of xenozoonosis. Xenotransplantation clinical programs, including monitoring of patients for PERV transmission, were slowly re-initiated but at that time the risk seemed to outweigh the benefits for the regulators and the investor community. Twenty years since, three different avenues of investigation have been pursued to decrease the severity of PERV infection and therefore improve safety of xenotransplantation.

1. New lines of research are identifying RT-inhibitors and developing antibodies that inhibit PERV infection [66]. It was also demonstrated that some licensed anti-retroviral drugs may be useful for controlling PERV infection in the unlikely event that xenotransplantation recipients show evidence of PERV infection [72].

2. The use of membrane-based extracorporeal systems to separate patient and pig cells further reduces the risk of PERV infection. This is illustrated by the results of an in-vitro study with a porcine-based bioartificial liver assist device.

The study was conducted to assess the presence and potential infectivity of PERV in the media collected from the HepatAssist System bioreactor containing either previously cryopreserved porcine hepatocytes or PK-15 cells [41]. Cell-free retrovirus produced by the PK-15 pig kidney cell line was shown to infect the human kidney 293 cell lines in vitro [64]. The supernatant consisted of the media surrounding the cells in the bioreactor; this was done to simulate plasma circulation during patient’s treatment. No evidence of PERV infection was observed in human 293 cells inoculated with either porcine hepatocyte supernatant, or 1000 fold concentrates of the medium circulating through the lumen of the fibers in the HepatAssist bioreactor. Results of the bioreactor containing PK-15 cells showed that the polysulfone hollow fiber membrane in the HepatAssist system decreased the risk of PERV transmission by a factor of 100,000. The study demonstrated the ability of a membrane to reduce risk of PERV transmission in a bioartificial organ thus increasing the product safety over the use of cells or organs without a membrane barrier.

3. The gene editing approach with the use of the CRISPR/Cas9 to target and inactivate a specific DNA sequence holds potential. In a recent study, Yang and colleagues [73] reported that they genetically engineered a one-step inactivation of 62 active PERV insertions and demonstrated a greater than 1000-fold reduction in PERV transmission to human cells. Much work lies ahead that includes generation and maintenance of viable pigs, and addressing concerns about off-target effects (inadvertent inactivation of non-target genes) [74]. However, this breakthrough technology showed that PERVs can be inactivated for clinical application. These results have reinvigorated the field of xenotransplantation.

##### Detectability

PERV detection methods have evolved, leading to improved assay sensitivity and specificity for detecting evidence of PERV transmission in patients receiving xenotransplantation therapies. Modern tools include methods of molecular virology based on PCR or other molecular biological methods measuring PERV-specific antibody responses [51]. As a result, the relatively low detectability in the mid-1990s has risen to level “medium” today.

##### Likelihood

When cross-species transmission of PERVs was discovered, there were no clinical data on PERV in patients exposed to pig organs, tissues or cells. In the past two decades, there has been a considerable number of publications concerning evaluation of PERV transmission to patients who were exposed to pig cells or tissues via bioartificial organs with or without the use of selective membranes and through direct perfusion of pig organs. Collectively, over 300 subjects have been monitored for PERV infection, and no PERV transmission was detected in any of these subjects through either clinical trials or through isolated human studies [41,42,44,45,75,76,77,78,79]. A summary of PERV test results in patients exposed to pig cells or tissues is presented in Table 1. The results show that there have been no instances of infection in humans. Based on this collective evidence of absence of PERV transmission, the likelihood of such infection has now evolved from being in the “high” category in the mid-1990, when data were unavailable but abundance of caution predominated, to the “medium” risk category at the present time.

##### Overall Risk Assessment

As discussed above, the risks of PERV infection have evolved. In the mid-1990s, both the potential for and likelihood of severe consequences of PERV transmission to humans were high and were therefore characterized as Class 1 in Table 2 (highlighted in red in the upper section). Taking into account also the low detectability of PERV infection, the PERV transmission was characterized as “high risk” (highlighted in red in the bottom section of Table 2).

Based on the current knowledge and observation of no evidence of PERV infection, the potential for PERV gene eradication, use of extracorporeal membrane-based bioartificial organs, and potential availability of anti-retroviral treatments, the severity of PERV transmission has decreased and can be assessed as “medium” (in blue font in the upper section of Table 3); the likelihood of PERV infection is also “medium” and therefore characterized as Class 2 in Table 3 (highlighted in blue in the upper section). Detectability has improved to “medium” as well, thus bringing the overall risk of PERV infection a level down and now characterized as being in the “medium risk” category (highlighted in blue in the bottom section of Table 3).

The goal of reducing the risk of PERV transmission is being attained.

## 5. Conclusions

Bioartificial organs are categorically needed to address organ shortage, which becomes more severe each year. Growing organs by employing decellularized scaffolds or 3D printing is both intriguing and promising; however, such technologies are still in their infancy and many hurdles remain such as cell sourcing, technology availability in case of emergency, manufacturability and delivery of bioartificial organs. In contrast, bioartificial organs that contain pig cells or tissues have gained considerable clinical experiences, and the attendant risks have been reduced. Because of PERV risk reduction and possible eradication, advances in gene editing to overcome immunological barriers, and lessons learned through clinical application, the future of closing the gap between organ demand and organ availability through the use of bioartificial organs looks more attainable than ever before.

Multiple barriers have been overcome, yet many remain to be addressed. As bioartificial organs provide a promising alternative to human organs for transplantation or regeneration, their translation to clinical applications has to be accelerated. Existing regulatory barriers for bioartificial organs employing pig cells are justified, but need to be aligned with current knowledge. Stability and steadiness of funding is crucial for conducting multi-center clinical trials.

While the risk of PERV infection, as we assessed in this paper, has currently evolved from the “high” category in the mid-1990s to the “medium” category, the risk of absence of commercially available therapies using bioartificial organs for life threatening diseases remains “high”.

## Figures and Tables

**Table 1:** Summary of porcine endogenous retrovirus (PERV) testing from patients exposed to bioartificial organs containing pig cells and to pig organs or tissues.

Targeted Disease Indication	BAO ^1^ System Name	Category of Xenotransplantation Product	Source of Cells/Tissues	Type of Exposure	Number of Patients	PERV Detected Yes/No	Reference
Liver failure	BLSS	Extracorporeal liver support system	Primary pig liver cells	Membrane bioreactor	5	No	[65]
	AMC-BAL	Extracorporeal liver support system	Primary pig liver cells	Membrane bioreactor	12	No	[41]
	RFB	Extracorporeal liver support system	Primary pig liver cells	Membrane bioreactor	7	No	[40]
	MELS	Extracorporeal hybrid liver support system	Primary pig liver cells	Membrane bioreactor	8	No	[36,37]
	HepatAssist	Extracorporeal liver support system	Cryopreserved pig liver cells	Membrane bioreactor	103	No	[33,34]
	–	Extracorporeal pig liver perfusion	Transgenic pig liver	Direct exposure	2	No	[66]
Chronic Glomerulonephritis	–	Extracorporeal pig kidney perfusion	Pig kidney	Direct exposure	2	No	[67]
Neurological conditions ^2^	–	Direct transplantation	Cells from fetal pigs	Direct exposure	24	No	[68]
Diabetes	DIABECEll^®^	Alginate-encapsulated cells	Porcine Islet cell Tx ^3^		16	No	[69,70]
	–		Porcine islet cell Tx ^3^	Direct exposure	10	No	[71]
Various indications	–	Extracorporeal pig organ perfusion, pig islets	Pig kidney, liver, spleen, islets ^4^	Direct exposure	160	No	[72]

^1^ Bioartificial Organs (BAO); ^2^ Ventral mesencephalon and lateral ganglionic eminence cells; ^3^ Transplantation (TX); ^4^ Patients treated with various pig tissues using different treatment modalities at different institutions for up to 12 years prior to sample collection and testing.

**Table 2:** PERV Risk Assessment based on scientific knowledge in mid-1990s ^1^.

**Severity**	**Likelihood**		
	**Low**	**Medium**	**High**
**High**	Class 2	Class 1	**Class 1**
**Medium**	Class 3	Class 2	Class 1
**Low**	Class 3	Class 3	Class 2
**Risk Class**	**Detectability**		
	**Low**	**Medium**	**High**
**Class 1**	High risk	High risk	Medium risk
**Class 2**	High risk	Medium risk	Low risk
**Class 3**	Medium risk	Low risk	Low risk

^1^ Layout of Table 2 and Table 3 is based on PDA Technical Report [80].

**Table 3:** PERV Risk Assessment based on the current scientific knowledge and clinical experience ^1^.

**Severity**	**Likelihood**		
	**Low**	**Medium**	**High**
**High**	Class 2	Class 1	Class 1
**Medium**	Class 3	Class 2	Class 1
**Low**	Class 3	Class 3	Class 2
**Risk Class**	**Detectability**		
	**Low**	**Medium**	**High**
**Class 1**	High risk	High risk	Medium risk
**Class 2**	High risk	Medium risk	Low risk
**Class 3**	Medium risk	Low risk	Low risk

^1^ Layout of Table 2 and Table 3 is based on PDA Technical Report [80].
